# Geometric Freezing of In-Situ Fibrillated PP/PA66 Composites via Low-Temperature Injection: Decoupling the Role of Draw Ratio and Compatibilization

**DOI:** 10.3390/polym18172049

**Published:** 2026-08-24

**Authors:** Run Zhang, Chenchao Fu, Guozheng Zhao, Weiheng Mo, Famin Zhao, Xiangrong Li, Qiuxu Chen, Lin Zhuo

**Affiliations:** 1 Guangxi Zhuang Autonomous Region Construction Engineering Quality Testing Center Co., Ltd., Nanning 530005, China; zhangrun_buct@163.com (R.Z.);; 2 College of Mechanical and Electrical Engineering, Beijing University of Chemical Technology, Beijing 100029, China

**Keywords:** in-situ microfibrillar composites, polypropylene/polyamide 66 blends, draw ratio, compatibilization

## Abstract

Controlling fibril generation and retaining their morphology during secondary thermal processing remain critical challenges for in-situ microfibrillar composites. Herein, we propose a “melt blending–hot drawing–low-temperature injection” cascade strategy to fabricate polypropylene/polyamide 66 (PP/PA66) composites. By decoupling the synergistic effects of draw ratio (λ) and compatibilizer (PP-g-MAH), an optimal fibrillation window was identified (15 wt% PA66, 3 wt% compatibilizer, λ = 9), which balances interfacial tension and viscous drag to form a dense, oriented microfibrillar network. This solid-state network accelerates matrix nucleation (though slightly restricting overall crystallinity) and induces gel-like rheological behavior through severe structural confinement. Crucially, we demonstrate that conventional high-temperature injection (265 °C) triggers Rayleigh instability, causing fibril break-up and mechanical degradation. Conversely, low-temperature injection (210 °C) successfully achieves the “geometric freezing” of the metastable fibril network. Consequently, the optimal composite exhibits maximized static strength (45.3 MPa) and a continuous, significant leap in notch impact toughness (10.16 kJ/m^2^). This work bridges the gap between flow-induced fibrillation and thermodynamic morphological retention, offering a robust physical mechanism for high-performance polyolefin composites.

## 1. Introduction

With the urgent demand for energy conservation and emission reduction in the transportation and aerospace sectors, the development of lightweight and high-strength polymer-matrix composites has become a core issue in materials science [1]. Polypropylene (PP), owing to its low density, excellent processing fluidity, and chemical stability, is widely regarded as an ideal matrix material for replacing metals [2]. However, PP itself is limited by a lower melt strength and significant notch sensitivity, making it difficult to directly meet the mechanical performance requirements of high-end structural components [3]. Although traditional glass fiber (GF) reinforcement technology can effectively improve rigidity, the high energy consumption of GF preparation, the wear and tear on processing equipment, and the difficulties in composite recycling contradict the current industrial trend of sustainable development [4,5,6]. Against this background, “in-situ fibrillation” technology has emerged [7]. This technology utilizes the deformation of immiscible polymer blends (such as PP/PA, PP/PET) under specific flow fields to generate reinforcing phase fibers with high aspect ratios in situ. Compared to externally added fibers, in-situ microfibrillar composites not only possess excellent potential for interfacial bonding but also exhibit outstanding isotropic reinforcement effects and recyclability, becoming a research hotspot for next-generation high-performance thermoplastic composites [8].

To obtain ideal high-aspect-ratio microfibrillar morphologies in immiscible polymer blends, academia has conducted extensive explorations into the “microfibril generation mechanism” [9,10]. Based on the classical Taylor droplet deformation theory, the fibrillation of the dispersed phase is controlled by the viscosity ratio, capillary number, and external processing flow field [10,11]. Recent studies have generally confirmed that, compared to traditional pure shear flow fields, strong extensional flow fields play a decisive advantageous role in inducing fibrillation [9,12]. Wang [13] established a dynamic prediction model describing the entire in-situ fibrillation process for the PP/PA6 blend system; by introducing the reduced capillary number to characterize the critical window for droplet deformation and breakup, the competitive effects of initial particle size and droplet coalescence on the final morphology were revealed. In the field of bio-based films, Dadouche et al. [14] proposed that when the critical capillary number criterion is met, the dispersed phase droplets could stably deform into nanofibers in an extensional flow field; this criterion provides a rheological basis for regulating in-situ micro/nano-fibrillation. However, the final topological structure of microfibrils is dually regulated by interfacial thermodynamics and processing dynamics. Huang et al. [15,16] utilized the alternating shear-extensional flow field provided by a tri-screw extruder (TTSE) to systematically verify the mechanism by which the PA66 dispersed phase undergoes a “deformation-coalescence-stretching” evolution to form high-aspect-ratio microfibrils, and found that the construction of the microfibril network transitioned the composite from liquid-like to gel-like behavior. On this basis, the introduction of interface engineering makes the fibrillation behavior more complex. Wang et al. [17] constructed a strong interfacial mechanical interlocking effect by introducing graphene oxide grafted polypropylene (PP-g-GO), which not only reduced interfacial tension but also significantly enhanced stress transfer efficiency through the “nodular” protrusions on the microfibril surface. Research by Cheng and Wang [18] further found that the non-uniform diffusion of compatibilizers at the interface led to the formation of concave-convex structures on the microfibril surfaces; such irregular microfibrils can effectively induce the matrix to form β-crystals and long-period lamellar stacks. Despite this, current research mostly focuses on the regulation of a single flow field or a single component. For the extremely immiscible PP/PA66 system, the non-linear coupling mechanism between the macroscopic extensional field (precisely controlled via draw ratio) and interfacial thermodynamic constraints (controlled via compatibilizer content)—especially how to decouple these two major factors to identify the “critical fibrillation window”—still lacks systematic experimental evidence and quantitative description.

However, existing studies have mostly focused on the “generation” of microfibrils during the extrusion stage, yet relatively neglected the “retention” challenge of microfibrils during secondary forming processes (such as injection molding). In-situ generated high-aspect-ratio microfibrils are essentially thermodynamically metastable structures. During subsequent high-temperature thermal processing (especially when the injection temperature exceeds the melting point of the dispersed phase), they are highly prone to spontaneous thermodynamic retraction and breakage (break-up behavior), leading to a drastic decline in the mechanical properties of the composites. For example, Jayanarayanan et al. [19] clearly pointed out in their study of the PP/PET microfibril system that, although a high draw ratio can effectively refine microfibrils, the PET microfibrils underwent significant thermodynamic retraction and breakage (break-up behavior) during the subsequent high-temperature injection molding process, resulting in a substantial loss of mechanical properties. Similarly, Kuzmanović et al. [20] found that when the injection temperature exceeded the melting point of the dispersed phase, the initially high-aspect-ratio microfibrils melted and coalesced, causing the flexural modulus of the samples to drop by about 17% and the impact performance to deteriorate significantly. Focusing on the thermal stability of microfibrillar structures, academia has established a solid-state geometric constraint strategy based on a “temperature window”. In the early stages of the microfibrillar composite concept, Refs. [21,22] laid the theoretical foundation through a drawing-annealing process: when the processing or annealing temperature was between the melting points of the two blend components, the high-melting-point component can maintain its solid-state oriented microfibrillar morphology, while the low-melting-point component rearranged into an isotropic matrix. This concept was verified in subsequent engineering practices; Liang et al. [23] found in the PP/PA6 system that strictly controlling the injection temperature between the melting points of PP and PA6 allowed some PA6 microfibrils to be retained in the matrix. However, for the PP/PA66 system, which has a greater polarity difference and is more difficult to fibrillate, existing literature mostly remains at the qualitative observation level.

Based on the aforementioned common challenges—namely, the unclear synergistic regulation laws of flow fields and interfaces during microfibril generation, and the morphological instability of microfibrils during secondary processing—this study prepared PP/PA66 in-situ microfibrillar composites using a “melt blending–tandem extrusion hot drawing–low-temperature injection” process. This work mainly explored the synergistic effects of the macroscopic draw ratio (λ) and the compatibilizer (maleic anhydride-grafted polypropylene, PP-g-MAH) content on the topological evolution of the “deformation-coalescence-fibrillation” of the PA66 dispersed phase, aiming to clarify the critical processing window during fibrillation. For the secondary forming stage, a low-temperature injection strategy between the melting points of the two phases is introduced to achieve the “geometric freezing” of the microfibrillar structure by leveraging the solid-state characteristics of the dispersed phase at the processing temperature; additionally, the effects of thermal processing history on morphological retention and mechanical properties were comparatively analyzed. Furthermore, this work systematically investigated the regulatory effects of the dense microfibril network on the crystallization dynamics of the matrix and the rheological properties of the melt, and analyzed the stress transfer and crack deflection mechanisms of the composites under impact loads combined with fracture mechanics models. The results aimed to establish the “process-morphology-rheology-property” correlation mechanism for the PP/PA66 system, providing experimental evidence and theoretical support for the preparation and application of high-performance in-situ microfibrillar composites.

## 2. Experiment & Methods

### 2.1. Experimental Materials, Equipment, and Testing Methods

Materials: In this study, polypropylene (PP, grade T30S) was selected as the continuous matrix phase, with a density of 0.91 g/cm^3^ and a melt flow index (MFI) of 3.0 g/10 min (230 °C, 2.16 kg), purchased from China Petroleum & Chemical Corporation, Beijing, China. The dispersed phase was polyamide 66 (PA66, grade FYR27, with a reported melt flow index of 9.0 g/10 min at 275 °C and 2.16 kg), with a density of 1.14 g/cm^3^, purchased from Pingdingshan Shenma Engineering Plastics Co., Ltd., Pingdingshan, China. To reduce the interfacial tension between the immiscible phases and enhance interfacial adhesion, maleic anhydride-grafted polypropylene (PP-g-MAH, grade CMG9801-GS, supplied by Dongguan Minglang Plastics Co., Ltd., Dongguan, China) with a grafting rate of approximately 1.0 wt% was introduced as a reactive compatibilizer.

Equipment: The melt blending stage employed a co-rotating twin-screw extruder (SHJ-20, Yangzhou Aichi Rubber & Plastic Machinery Co., Ltd., Gaoyou, China) with a length-to-diameter (L/D) ratio of 40:1. A custom-built hot drawing traction device equipped with a precise temperature control and variable frequency speed regulation system was used. Sample molding was performed using a Haitian MA1200 injection molding machine, China, Ningbo. Microstructural observation was conducted using a Hitachi S-4800 scanning electron microscope (SEM, Tokyo, Japan). Rheological properties were tested with a TA Instruments ARES-G2 rotational rheometer. Thermal properties were analyzed using a TA Q20 differential scanning calorimeter (DSC, New Castle, DE, USA). Mechanical property testing was completed on an electronic universal testing machine (KXWW-20C) and an Izod impact testing machine (KXJJ-50A) from Chengde Taiding Co., Ltd., Chengde, China.

### 2.2. Processing Procedure

Prior to processing, the PP, PA66, and PP-g-MAH pellets were thoroughly dried in a vacuum drying oven (DZF-6050, Beijing Luxi Technology Co., Ltd., Beijing, China) at 80 °C for 24 h to rigorously remove moisture and prevent polymer hydrolysis. The moisture content of the polymers was not measured. The dried materials were pre-mixed in a high-speed mixer for 10 min according to the designed ratios and fed into a co-rotating twin-screw extruder (TSE, SHJ-20, Yangzhou Aichi Rubber & Plastic Machinery Co., Ltd., Gaoyou, China) with a length-to-diameter (L/D) ratio of 40:1. The TSE melt blending and pelletizing were conducted with a barrel temperature profile of 170 °C, 220 °C, 250 °C, and 275 °C for Zones 1–4, maintained at 275 °C from Zone 5 to the die (Zone 9), and a screw speed of 150 rpm. The primary blended extrudates were then water-cooled and pelletized.

**Figure 1:**
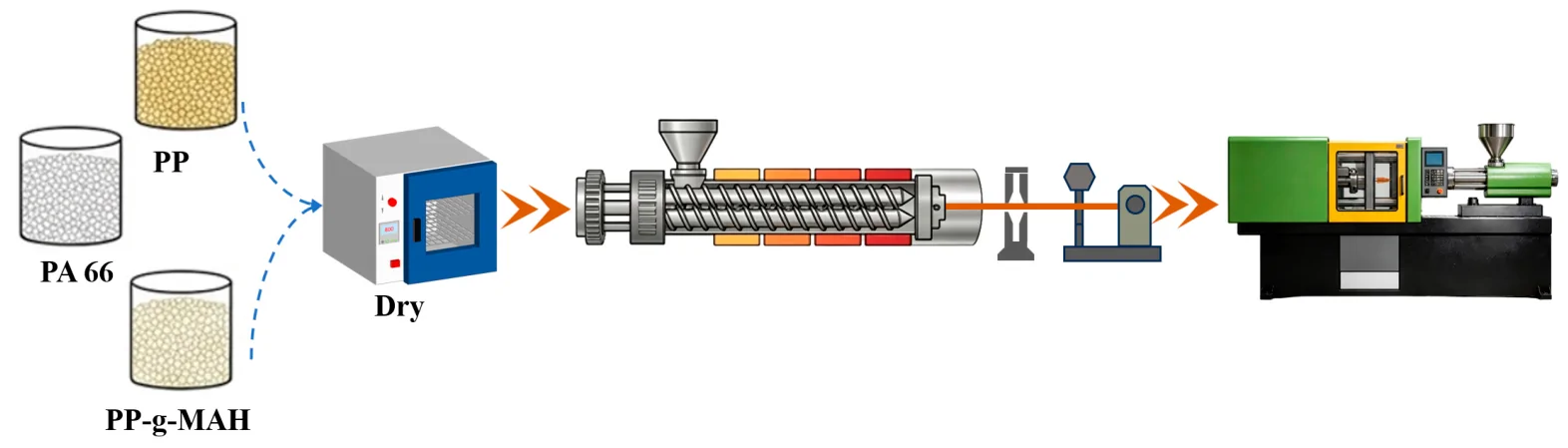
Schematic diagram of experimental process.

The blended pellets were re-dried and subsequently subjected to a secondary extrusion coupled with an in-line hot drawing process. The materials were fed back into the same TSE, operating at a lower barrel temperature profile: 170 °C, 190 °C, and 245 °C for Zones 1–3, and maintained at 245 °C from Zone 4 to the die (Zone 9), with a screw speed of 35 rpm. Immediately upon exiting the die, the continuous melt strands were cooled in a water bath and subjected to a uniaxial hot drawing stress field using a custom-built variable-frequency traction device (manufactured by Beijing Paigu Precision Machinery Co., Ltd., Beijing, China). The draw ratio (λ, ranging from 1 to 9) was precisely controlled by adjusting the traction speed. The stretched microfibrillar composite strands were rapidly solidified and subsequently pelletized for injection molding. The schematic process is shown in Figure 1.

Finally, the pelletized microfibrillar composite strands were molded into test specimens using a Haitian MA1200 injection molding machine. To achieve the “geometric freezing” of the metastable PA66 microfibrils, a low-temperature injection strategy was applied. After a 2-h preheating process, the injection molding temperature profile from the feeding zone to the nozzle was strictly maintained at 170 °C, 190 °C, 210 °C, and 205 °C, with the mold temperature set at 50 °C. The maximum melt temperature was precisely controlled at 210 °C, which is above the melting point of PP but below that of PA66. The melt was injected into a standard mold cavity to form dumbbell-shaped specimens (geometrical dimensions: 150 × 10 × 4 mm^3^) or tensile and flexural tests. The main injection molding parameters included an injection pressure of 50 MPa and a holding time of 60 s.

### 2.3. Property Characterization

SEM: The cross-sectional morphology was observed using a field emission scanning electron microscope (SEM, S-4800, Tokyo, Japan). In order to maintain the original internal structure and avoid plastic deformation during the cutting process, the sample was immersed in liquid nitrogen and subjected to low-temperature brittle fracture. Before conducting SEM observation, a thin layer of gold was sputtered onto the fractured surface of the sample using an ion sputtering apparatus to improve its conductivity and eliminate charge accumulation effects.

Tensile and flexural property testing: At a speed of 10 mm/min, five standard dumbbell-shaped injection-molded specimens (dimensions: 150 mm in length, 10 mm in width, and 4 mm in thickness) were selected. The tensile strength test was conducted at a room temperature of 25 °C according to the GB/T 1040.2-2006 standard [24], with a required span length of S = 100 mm, using an electronic universal testing machine (KXWW-20C, Chengde Taiding Testing Machine Manufacturing Co., Ltd., Chengde, China). The flexural strength and modulus tests were conducted according to the GB/T 9341-2008 standard [25], with a required span length of S = 64 mm, using an electronic universal testing machine (KXWW-20C, Chengde Taiding Testing Machine Manufacturing Co., Ltd., Chengde, China).

Notch impact strength: Five injection-molded specimens were selected and notched using a notch preparation machine, narrowest width Bn of the notch = 2 mm, and bottom radius Rn of the notch = 0.25 mm. The impact property test was conducted according to the GB/T 1843-2008 standard [26] using an Izod impact testing machine (KXJJ-50A, Chengde Taiding Testing Machine Manufacturing Co., Ltd., Chengde, China).

Differential Scanning Calorimetry (DSC): Thermal behavior was analyzed using a differential scanning calorimeter (DSC TA Q20, New Castle, DE, USA). The samples underwent a heat-cool-heat cycle from 20 °C to 280 °C, then back to 20 °C, and finally heated again to 280 °C, with both heating and cooling rates set at 10 K/min. The thermal data were collected and analyzed from the second heating scan to eliminate thermal history. The melting enthalpy (∆Hm) of the material is tested, and the crystallinity is calculated using Formula (1) [27].

(1) $$\begin{array}{c} X_{c}=\frac{\Delta H_{x}}{\Delta H_{x}^{0}\times \omega_{x}}\times 100\% \end{array}$$

where ${∆H}_{x}$ represents the melting enthalpy of polymer *X*; $\omega_{x}$ is the weight percentage of the polymer in the composite; ${∆H}_{x}^{0}$ is the melting enthalpy of the completely crystalline polymer, where ${∆H}_{PP}^{0}$ is 209.2 J/g [28], ${∆H}_{PA66}^{0}$ is 195.69 J/g [29].

Melt Flow Rate (MFR): The MFR was measured using a melt flow indexer (RL-Z1B1, Shanghai Sida Scientific Instrument Co., Ltd., Shanghai, China) according to the GB/T 3862-2000 standard [30]. The test was performed at 230 °C with a load of 2160 g. After steady melt flow was achieved, extrudate samples were collected and weighed every 30 s. This procedure was repeated five times, and the average value was calculated.

Rheological property testing: Rheological measurements were conducted using a rotational rheometer (TA ARES-G2, Waters, New Castle, DE, USA) equipped with 20 mm diameter parallel plates and a 1 mm gap. The test was performed at a temperature of 195 °C with an angular frequency sweep range set from 0.1 to 100 rad/s.

Fourier Transform Infrared Spectroscopy (FTIR): Testing was performed using a Nicolet 6700 FTIR with a resolution of 4 cm^−1^. Individual components and composites were analyzed by taking the average spectrum of 16 scans over a scanning range of 500–4000 cm^−1^.

## 3. Results and Discussion

### 3.1. Synergistic Control of Microscopic Morphology by Extensional Flow Field and Interface Engineering

Microscopic morphology is the core factor determining the performance of in-situ microfibrillar composites. This section systematically analyzed the regulatory laws of dispersed phase content, compatibilizer content, and draw ratio on the morphology of the PA66 dispersed phase.

#### 3.1.1. Effect of Dispersed Phase Content on Microfibril Morphology

**Figure 2:**
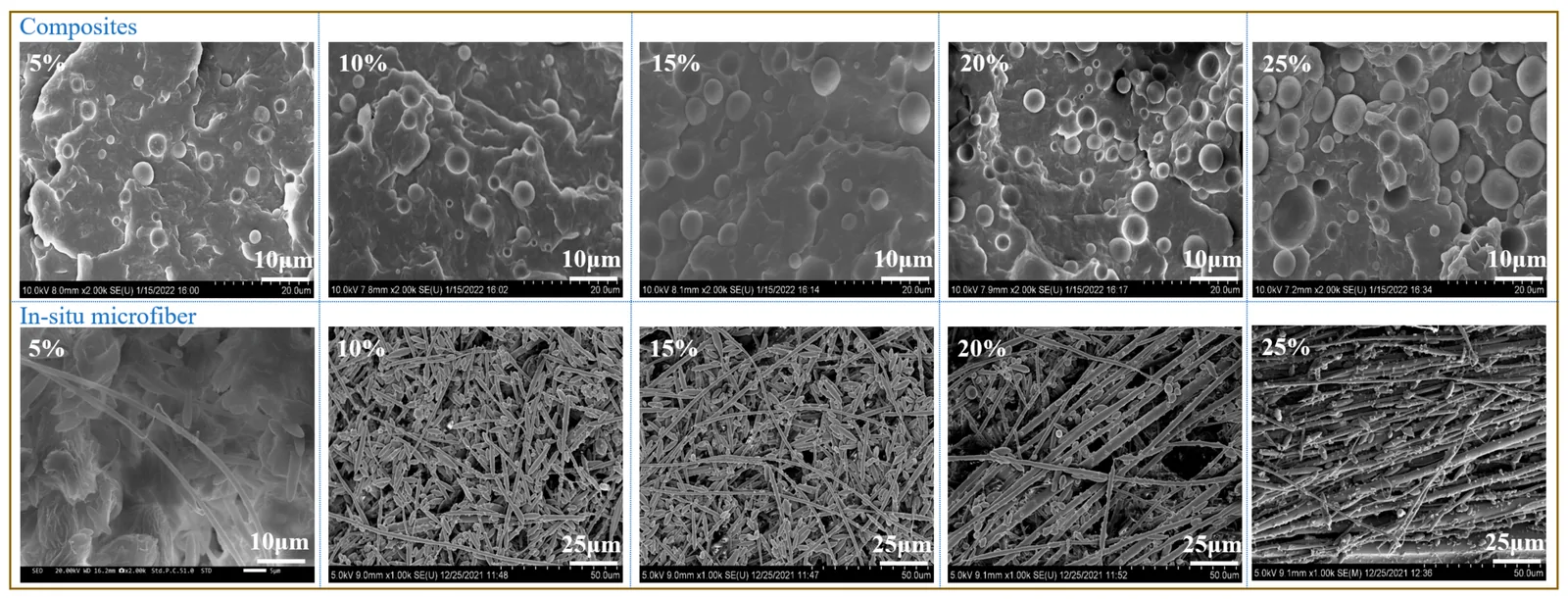
SEM images of PP/PA66 composites with different dispersed phase contents and in-situ microfibers.

Figure 2 shows the SEM images of the composites and in-situ microfibrils for PP/PA66 with different dispersed phase contents. It can be seen that in the undrawn PP/PA66 blends, PA66 is dispersed as spherical droplets in the PP matrix with clear two-phase interfaces, proving that PP and PA66 are thermodynamically immiscible, which is consistent with literature reports [31,32]. As the PA66 content increased from 5% to 25%, both the number and size of the dispersed phase spherical droplets simultaneously increased; this was because, at high contents, dispersed phase droplets were prone to collision and coalescence, forming larger-sized particles.

After hot drawing at a draw ratio of λ = 4, the PA66 dispersed phase transformed from spherical droplets to oriented microfibrils, achieving in-situ fibrillation. When the PA66 content was 5%, only a small amount of the dispersed phase formed microfibrils with small aspect ratios, while the majority remained as ellipsoidal particles; this was because the small size of the dispersed phase droplets at low contents made continuous deformation difficult in the extensional flow field. As the PA66 content increased to 15%, the number of microfibrils formed by the dispersed phase significantly increased, the aspect ratio greatly improved, and the distribution was uniform. When the PA66 content exceeded 15%, the microfibril diameter increased, and agglomeration was prone to occur. This result aligns with the Taylor droplet deformation theory [33,34]: when the dispersed phase content is moderate, the viscous drag force exerted on the droplets in the extensional flow field exceeds the interfacial tension, leading to continuous deformation and the formation of microfibrils; when the content is too high, droplet coalescence results in excessively large sizes, increasing deformation difficulty and leading to fiber agglomeration.

#### 3.1.2. Effect of Compatibilizer Content on Microfibril Morphology

**Figure 3:**
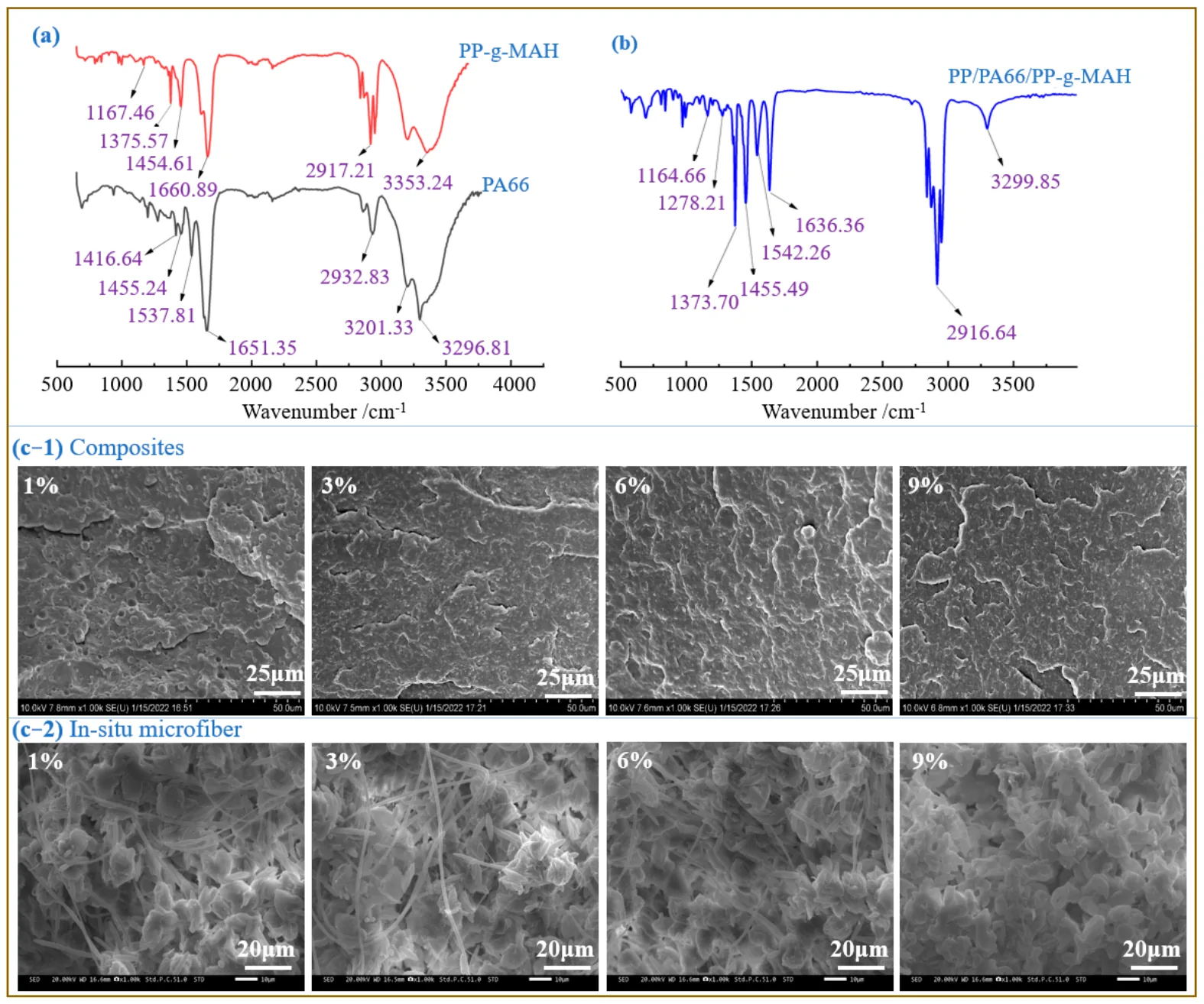
Infrared spectra of (**a**) PA66 and PP-g-MAH and (**b**) PP/PA66/PP-g-MAH. (**c-1**,**c-2**) SEM images of PP/PA66 composites with different compatibilizer contents and in-situ microfibers.

The addition of PP-g-MAH significantly altered the interfacial interactions between the PP and PA66 phases and the dispersed phase morphology. In the undrawn blends, as the PP-g-MAH content increased from 1% to 9%, the size of the PA66 spherical droplets gradually decreased, and their distribution in the matrix became more uniform. This was because the maleic anhydride groups in PP-g-MAH underwent a grafting reaction with the amide groups of PA66, forming a PP-g-PA66 graft copolymer at the two-phase interface, which reduced the interfacial tension and improved interfacial compatibility. This conclusion was also verified by the FTIR test results (Figure 3a,b): a characteristic absorption peak of the C-N-C functional group appeared at 1542.26 cm^−1^ in the composite material, proving the occurrence of the grafting reaction.

The results after hot drawing show a critical effect of the compatibilizer content on fibrillation: when the PP-g-MAH content was 3%, the microfibrils formed by PA66 had finer diameters, larger aspect ratios, and significantly better distribution uniformity than the samples without the compatibilizer. When the compatibilizer content reached 9%, PA66 could not form continuous microfibrils. The core mechanism was as follows: a low compatibilizer content reduced the interfacial tension, making the dispersed phase droplets more prone to deformation in the extensional flow field, while simultaneously enhancing the interfacial adhesion, preventing slippage between the two phases during drawing, and promoting microfibril formation. Conversely, when the compatibilizer content is too high, the excessively strong interfacial adhesion restricted the deformation and orientation of the dispersed phase droplets, which was detrimental to microfibril formation. This study clearly identified this critical compatibilizer content for fibrillation in this system for the first time.

#### 3.1.3. Effect of Draw Ratio on Microfibril Morphology

**Figure 4:**
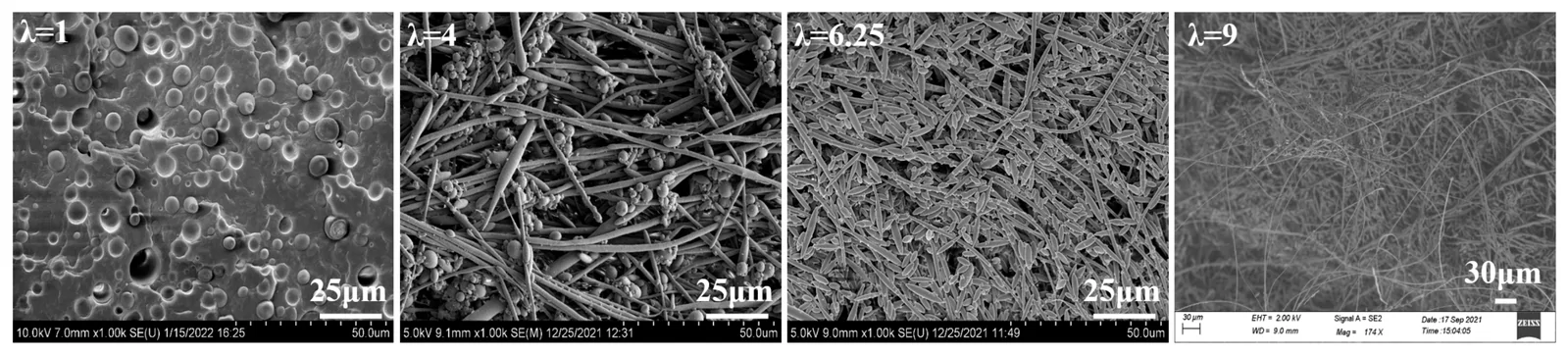
SEM images of PP/PA66 (85/15) in-situ microfiber composites under different stretching ratios.

The draw ratio is the core parameter for regulating microfibril dimensions. As can be seen from the microstructural images in Figure 4, when the draw ratio is λ = 1 (undrawn), PA66 remained as spherical droplets with no fibrillation occurring. As the draw ratio increased from 1 to 9, the number of PA66 microfibrils continued to increase, the average diameter continuously decreased, the aspect ratio significantly improved, and the degree of orientation steadily increased. The physical essence of this phenomenon was that as the draw ratio increased, the axial tensile stress and viscous drag force acting on the dispersed phase droplets synchronously increased, causing the droplets to be continuously stretched and thinned. Small-sized droplets that were originally unable to deform also underwent coalescence and deformation to form continuous microfibrils, achieving precise regulation of the microfibrillar morphology.

### 3.2. Effect of Microfibrillar Structure on the Crystallization and Rheological Behavior of Composites

This section analyzes the effects of the microfibrillar structure on the matrix crystallization behavior and melt rheological behavior of the composites at the mesoscopic level.

#### 3.2.1. Analysis of Crystallization Behavior

**Figure 5:**
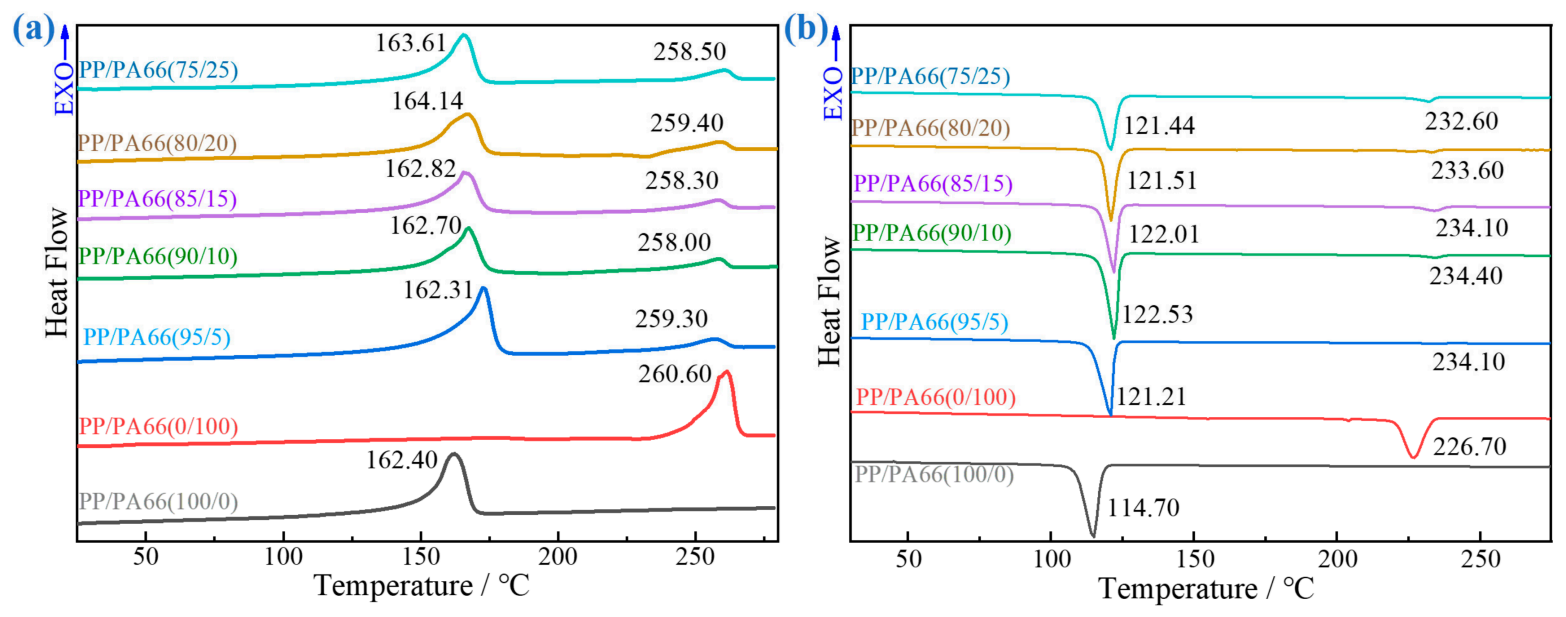
(**a**) Melting curves of PP/PA66 composite samples with different dispersed phases of PA66 and (**b**) the corresponding crystallization curves.

The DSC test results (Figure 5, Table S1) reveal the profound impact of the microfibrillar network on the thermal behavior of the composites. First, regarding the crystallization temperature (Tc), a significant change was observed between the neat polymer and the blends. The Tc of pure PP is 114.70 °C. After adding PA66 and achieving in-situ fibrillation, the Tc of the PP matrix abruptly increased to above 121 °C (reaching 121.21 °C even at only 5 wt% PA66). This substantial shift indicated that the solid PA66 microfibrils act as highly efficient heterogeneous nucleating agents, lowering the nucleation energy barrier for the PP matrix. Interestingly, as the PA66 content further increased from 5 wt% to 25 wt%, the Tc of the PP matrix did not exhibit any significant change, remaining relatively stable between 121.21 °C and 122.53 °C. This implied that a relatively low content of microfibrils is already sufficient to provide saturated heterogeneous nucleation sites for the matrix.

Most importantly, the overall crystallinity (Xc) of the PP matrix in all composites (ranging from 40.82% to 53.32%) remained consistently lower than that of pure PP (55.69%). This change demonstrated that while microfibrils promoted early nucleation, the solid PA66 dispersed phase fundamentally acted as a physical barrier. It restricted the mobility and regular folding of PP molecular chains, thereby inhibiting the overall extent of crystal growth. Furthermore, the Xc values exhibited a distinct composition-dependent trend. As the PA66 content increased from 10 wt% to 20 wt%, the crystallinity of PP showed a gradual recovery and upward trend, suggesting that the increase in nucleation sites slightly offset the barrier effect. However, when the PA66 content reached 25 wt%, the Xc slightly decreased to 53.17%. This confirmed that excessive microfibrils formed an overly dense intertwined network, confining the movement space of PP molecular chains and dominating the hindrance of further crystal growth. Finally, from the melting curves, it can be seen that two independent melting peaks corresponding to PP and PA66 exist simultaneously in the composite material, confirming the thermodynamic immiscibility of these two polymers.

**Figure 6:**
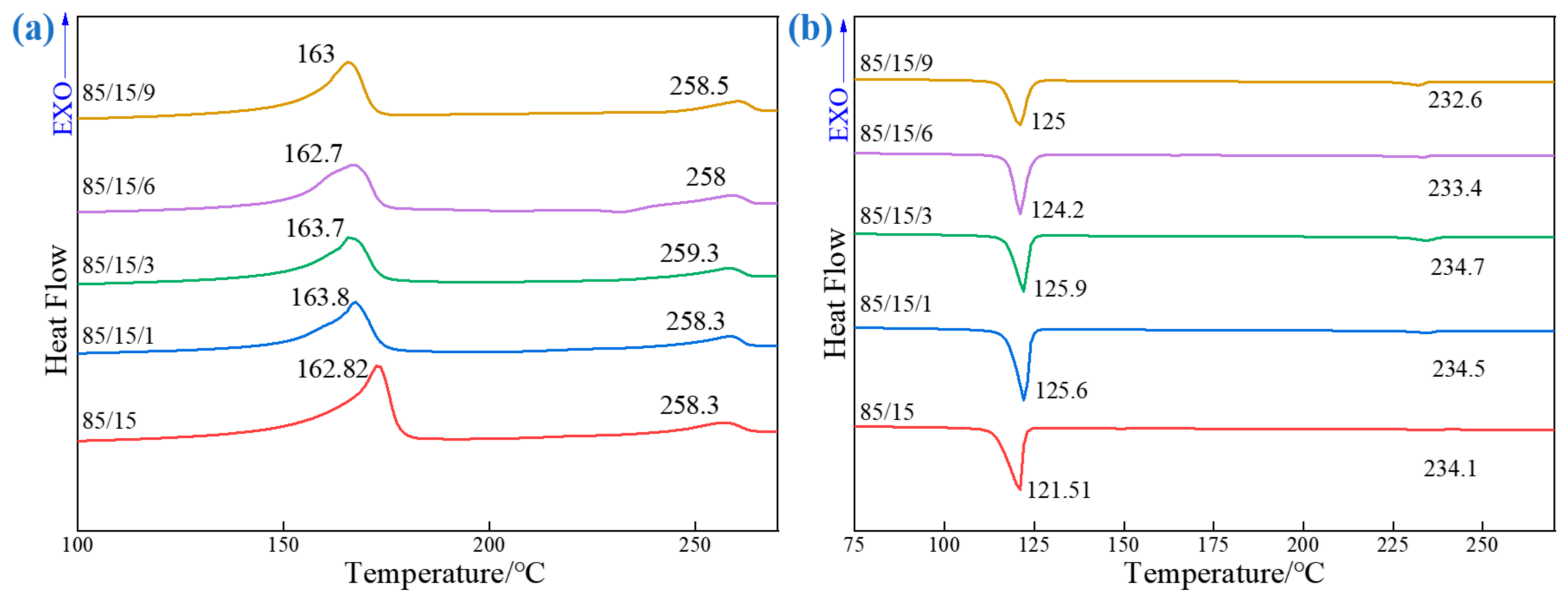
(**a**) Melting curves of PP/PA66 in-situ microfiber composites with different compatibilizer contents and (**b**) the corresponding crystallization curves.

The introduction of the compatibilizer PP-g-MAH introduced further, and highly significant, structural variations to the composites (Figure 6, Table S2). Similar to the trend observed with varying PA66 contents, a clear distinction must be made between actual thermal shifts and experimental variations. When comparing the uncompatibilized system (PP/PA66 = 85/15) to the compatibilized ones, a significant step-wise increase in the crystallization temperature of the PP phase is immediately evident upon the initial addition of the compatibilizer (jumping from 121.51 °C to approximately 125 °C). This confirmed an enhanced nucleating efficiency. However, as the compatibilizer loading varies from 1 wt% to 9 wt%, the resulting minor fluctuations in Tc (between 124.20 °C and 125.90 °C) are not statistically significant and fall within standard DSC experimental error.

Instead, the most critical and significant change induced by the compatibilizer was the substantial improvement in actual crystallinity. The Xc of the PP matrix steadily and significantly increased from 48.03% (in the uncompatibilized blend) to 52.78% (at 1 wt%), reaching a maximum of 58.81% (at 9 wt%). As discussed previously, uncompatibilized PA66 microfibrils acted as a physical barrier that restricted PP chain folding. The addition of the compatibilizer effectively overcame this limitation. By significantly reducing interfacial tension, the compatibilizer refined the PA66 microfibril diameter (as corroborated by previous SEM observations), creating a much denser network with an exponentially larger specific surface area. This refined topology provided a vast abundance of highly efficient nucleation sites, which outcompeted the steric hindrance and facilitated a higher overall degree of crystallization in the PP matrix.

Finally, for the dispersed PA66 phase, the compatibilizer restricted its chain mobility. The glass transition temperature of PA66 shifts gradually to higher temperatures (from 50.00 °C to 62.10 °C), and its crystallization exothermic peak weakened slightly. This indicated that strong interfacial interactions and in-situ formed PP-g-PA66 copolymers constrained the molecular chain arrangement of PA66 at the phase boundary.

#### 3.2.2. Rheological Behavior and Melt Flow Properties Analysis

**Figure 7:**
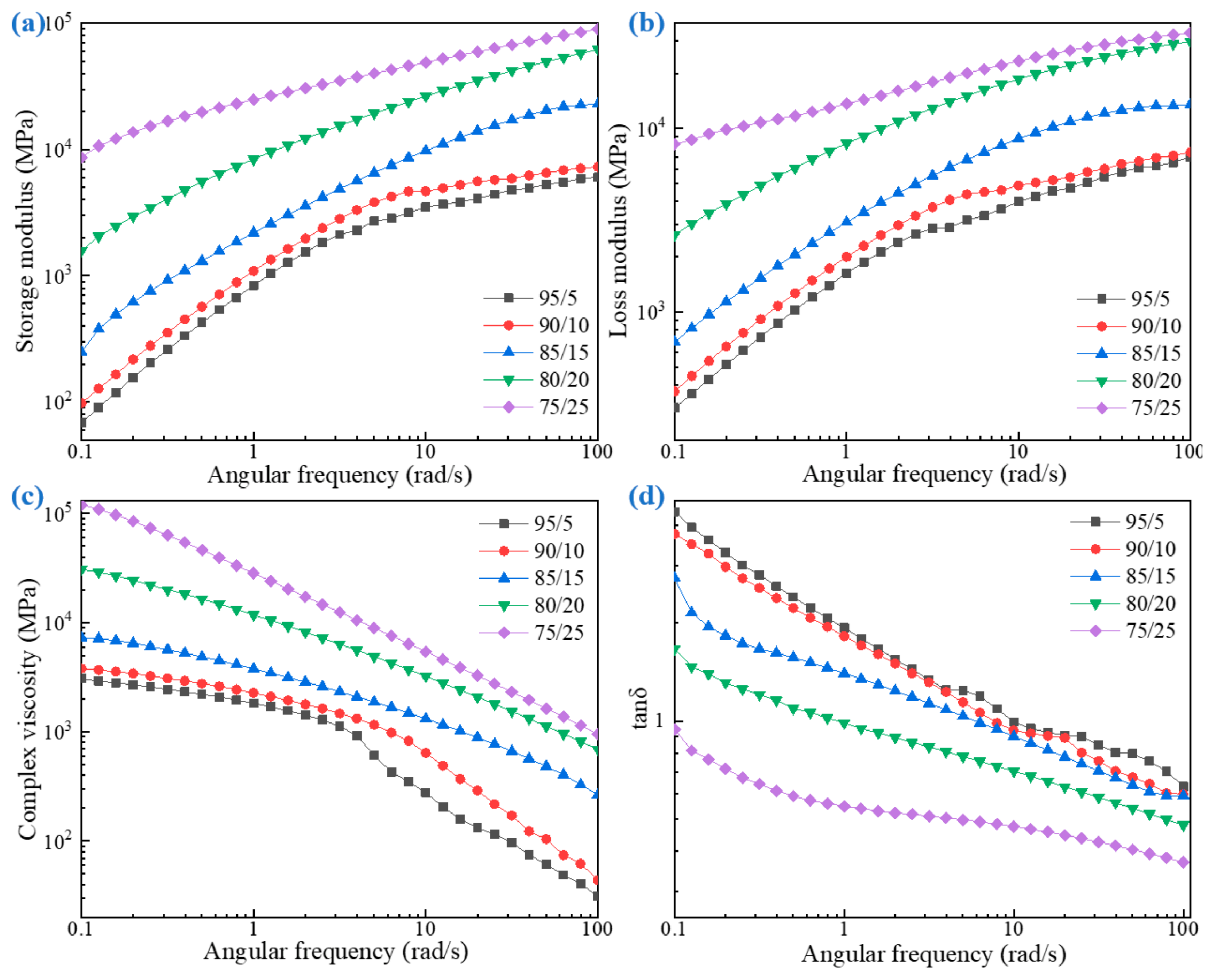
The relationship between (**a**) storage modulus, (**b**) loss modulus, (**c**) complex viscosity, (**d**) loss factor and angular frequency of PP/PA66 in-situ microfiber composites with different dispersed phase contents.

The dynamic rheological property test results of the composites reflected the physical characteristics of the microfibril network in the melt state. Both the storage modulus (G′) and loss modulus (G″) increased linearly with increasing angular frequency (ω) (Figure 7). At the same ω, as the PA66 content increased from 5 wt% to 25 wt%, G′ and G″ improved significantly.

It is crucial to note that at the rheological testing temperature of 195 °C (and MFR testing at 230 °C), the PP matrix is fully molten, whereas the PA66 (Tm ≈ 260 °C) remained completely in a solid state. Therefore, the observed rheological changes were not caused by the blending of two polymer melts with different viscosities, but were entirely dominated by the structural hindrance of the solid microfibers. As the PA66 content increased, these rigid, solid microfibrils intertwined to form a progressively denser three-dimensional physical network. This dense geometric skeleton imposed a severe confinement effect on the relaxation and movement of the PP molecular chains, exponentially enhancing the melt’s ability to resist deformation.

The complex viscosity (η*) of all samples decreased with increasing ω, exhibiting typical pseudoplastic fluid characteristics; furthermore, higher PA66 contents resulted in more pronounced shear-thinning phenomena. The trend of the loss factor (tan δ) decreasing with increasing PA66 content further confirms the gel-like rheological response induced by the microfibril network. The evolution law of the MFR further quantified the impact of the microscopic structure on processing performance.

**Figure 8:**
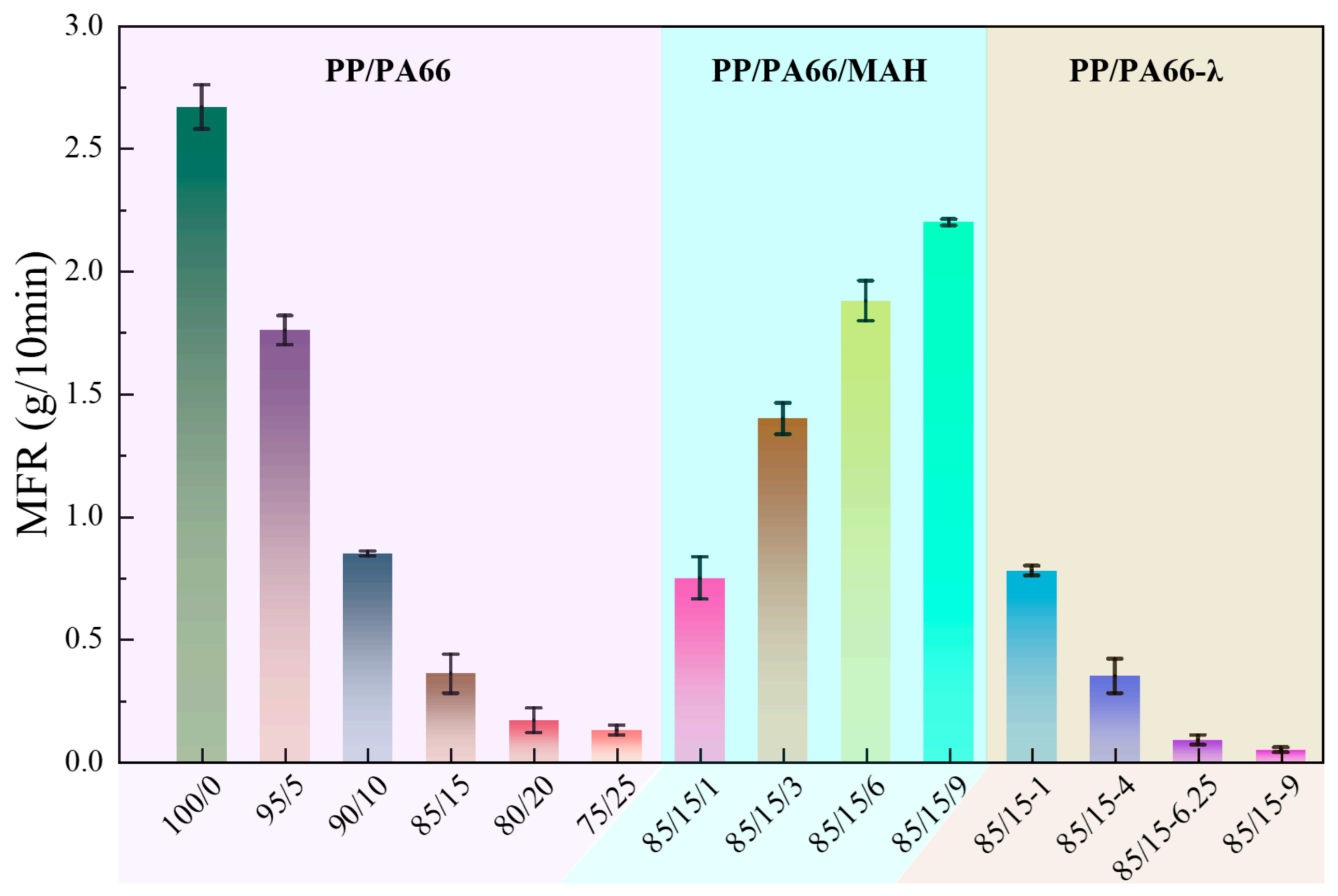
MFR of PP/PA66 in-situ microfiber composites with different PA66 contents.

The MFR values of all composite materials were lower than that of pure PP (2.8 g/10 min), reflecting the hindrance effect of the solid PA66 microfibrils on the flow of the matrix (Figure 8). As the PA66 content increased or the draw ratio (λ) rose from 1 to 9, the MFR showed a monotonic downward trend, reaching its lowest point at λ = 9 due to the formation of a denser intertwined network. However, the addition of the compatibilizer had a reverse regulatory effect on the MFR. As the PP-g-MAH content increased from 1 wt% to 9 wt%, the MFR rebounded from lower values and reached its maximum at 9 wt%. The physical essence of this phenomenon is that the compatibilizer refined the microfibril diameter and improved interfacial wettability, making the microfibrils and the PP matrix bind more tightly together, which thereby reduced the flow resistance at the interface and optimized the macroscopic processing fluidity of the system.

### 3.3. Macroscopic Mechanical Leap and Fracture Mechanisms

**Figure 9:**
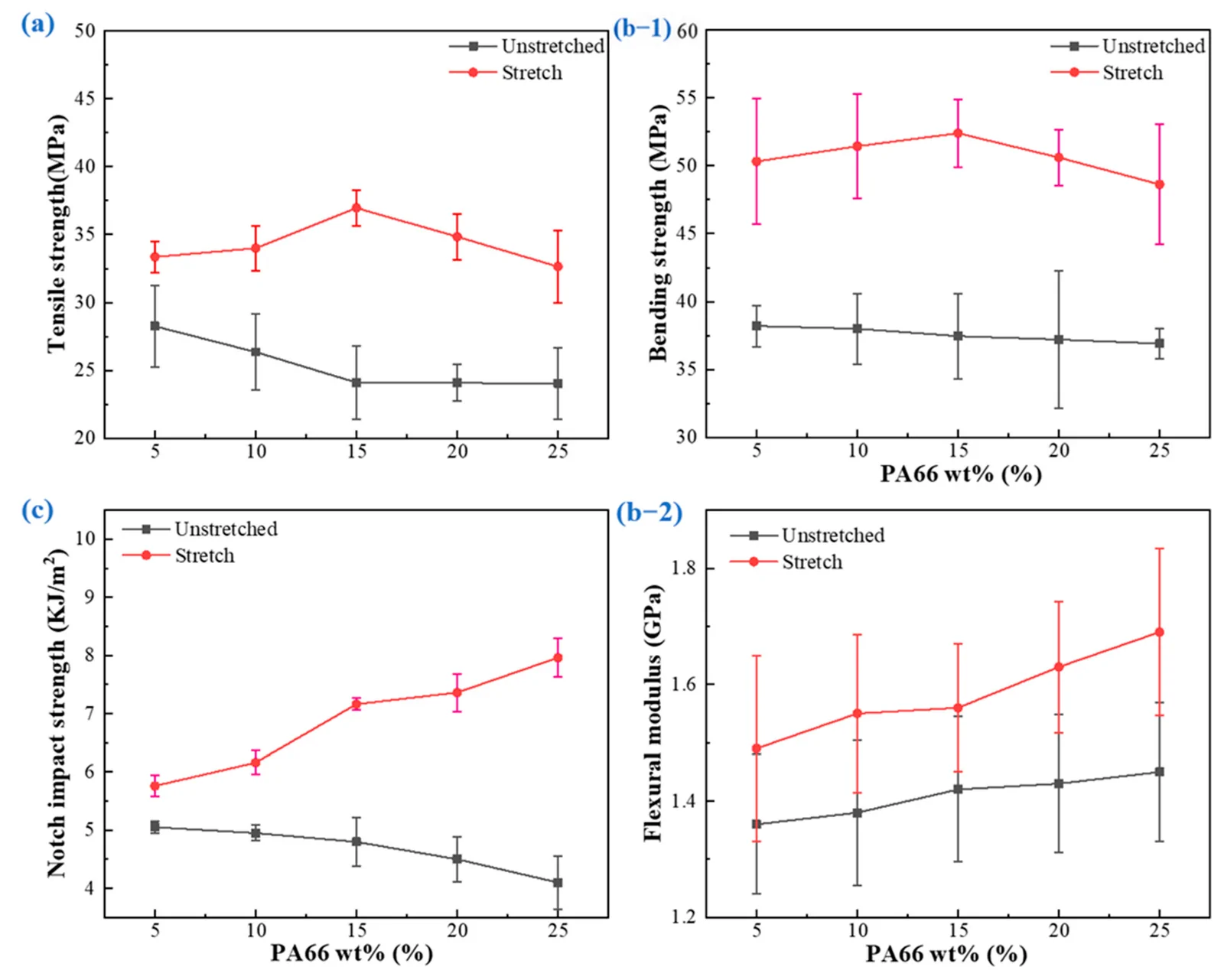
Tensile properties of PP/PA66 in-situ microfiber composites with different PA66 contents (**a**); (**b-1**,**b-2**) Bending performance; (**c**) The impact of impact performance.

The tensile and flexural mechanical properties of in-situ microfibrillar composites fundamentally depend on the efficiency of external load transfer from the flexible matrix to the high-modulus microfibrillar skeleton. The mechanical property test results show (Figure 9) that the tensile strength of the undrawn PP/PA66 blends continuously decreased with the increase in the dispersed phase PA66 content. This was mainly attributed to the fact that the dispersed phase in the immiscible system assumed a spherical droplet morphology, lacking effective stress transfer capability, and as the content increased, interfacial voids and other defects multiplied to form stress concentration sources. Conversely, after secondary extrusion and hot drawing fibrillation, the mechanical properties of the stretched composite materials were significantly greater than those of the unstretched samples. However, when taking the error bars (standard deviations) into consideration, the tensile and flexural strengths of the stretched composites show no statistically significant change across different PA66 contents (ranging from 5 wt% to 25 wt%), as the minor variations fell entirely within the margins of experimental error. This lack of change was not unexpected, as the tensile and flexural properties of polymer systems were generally much less sensitive to changes in microscopic morphology and processing conditions compared to the Izod impact strength. In distinct contrast, the notch impact strength exhibited a statistically significant and continuous improvement with higher PA66 contents. By directly correlating these macroscopic tensile test results with the SEM morphological observations (Figure 2 and Figure 4), the underlying micromechanical role of the dispersed phase became clear. The formation of the highly oriented microfibrillar network primarily acted to dissipate impact energy through fiber deformation and crack deflection. Meanwhile, the static tensile strength reaches a plateau because the structural reinforcement provided by the microfibers is dynamically balanced by the simultaneous increase in interfacial defects (voids) at higher dispersed phase contents.

**Figure 10:**
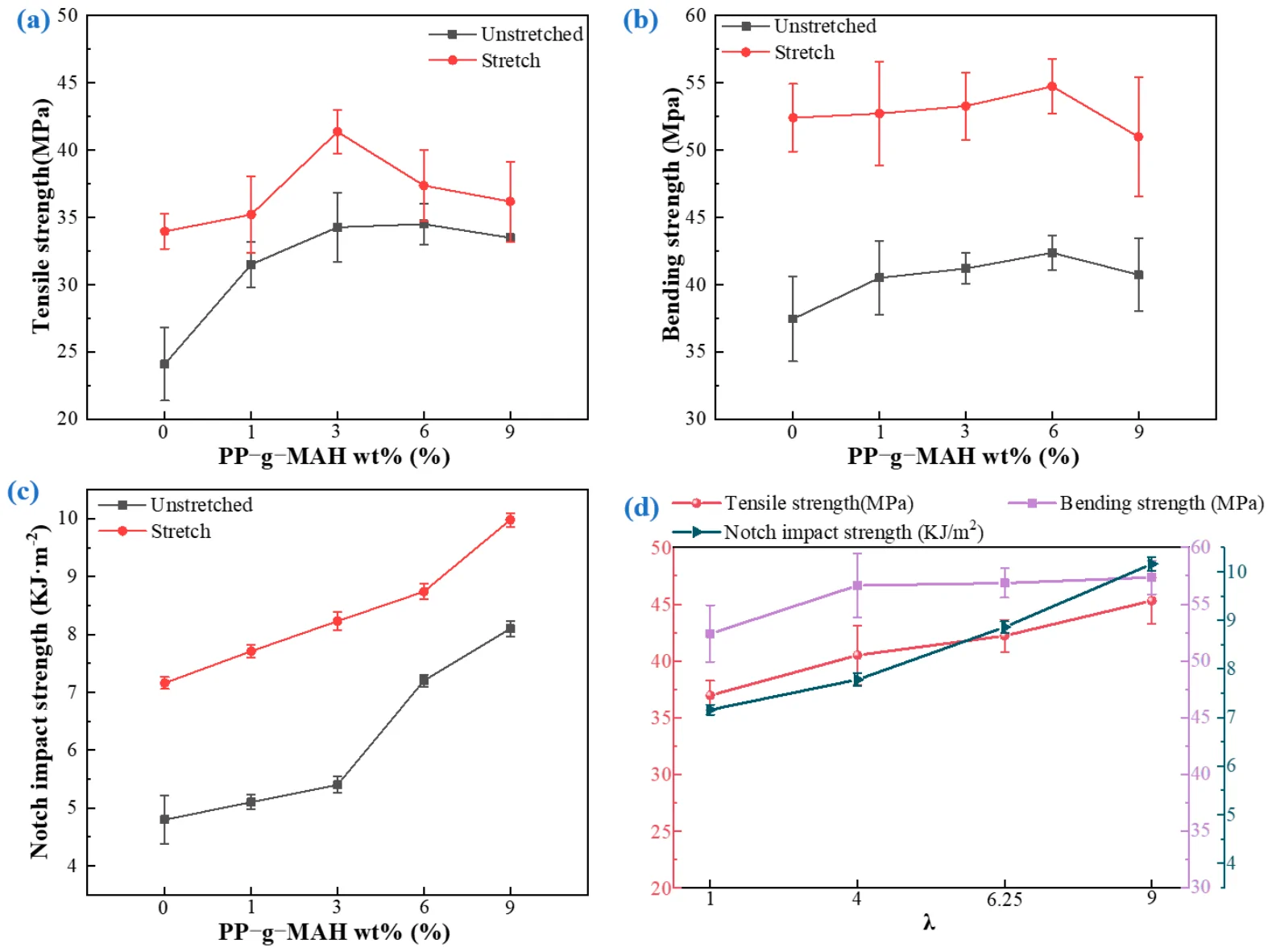
Shows the (**a**) tensile strength, (**b**) flexural strength, and (**c**) notch impact strength of PP/PA66 composites with different compatibilizer contents. (**d**) The influence of different stretching ratios on the mechanical properties of PP/PA66 (85/15) microfiber composites.

The introduction of interface engineering further regulated the mechanical behavior and stress transfer mechanism of the composite system. After adding the PP-g-MAH compatibilizer, the mechanical indicators of the composites exhibited varying degrees of change (Figure 10a–c). When considering the error bars, the data revealed that the addition of the compatibilizer improved the tensile and flexural strengths compared to the uncompatibilized system; however, across the compatibilizer contents from 1 wt% to 9 wt%, the fluctuations largely fell within experimental error, indicating a performance plateau. Similar to the effect of the dispersed phase content, this demonstrated that these static tensile and flexural properties were relatively insensitive to further interfacial modifications. This indicated that an appropriate amount of compatibilizer effectively improved the compatibility between the matrix and the dispersed phase, strengthened the interfacial adhesion, and formed a robust microfibrillar skeleton with a higher aspect ratio under the extensional flow field, thereby optimizing the stress transfer efficiency. Unlike the relatively stable tensile and flexural strengths, the notched impact strength of the composite material showed a statistically significant, monotonic positive dependence on the compatibilizer content. When the compatibilizer content reached 9 wt%, the notched impact strength of the system improved to 9.98 kJ/m^2^. This was primarily attributed to the fact that extremely strong interfacial adhesion significantly increased the fracture energy required for interfacial debonding, allowing the material to dissipate more energy through microscopic interfacial deformation when subjected to an instantaneous impact load.

The draw ratio (λ), as a critical kinematic parameter determining the geometric topological structure of the microfibrils, plays a dominant role in the final macroscopic toughness and fracture behavior of the material. As shown in Figure 10d, as the draw ratio increases from 1 to 9, the tensile strength of the PP/PA66 (85/15) composite almost linearly increases from 36.96 MPa to 45.3 MPa, and the notched impact strength increased from 7.16 kJ/m^2^ to 10.16 kJ/m^2^. In systems with a spherical dispersed phase formed without drawing or at low draw ratios, cracks propagating through the material easily penetrated the dispersed droplets directly or induced interfacial debonding, forming a linear propagation path with low energy consumption. In contrast, within the microfibrillar network system induced by a high draw ratio extensional flow field, the intertwining high-aspect-ratio fibers act as bridges for stress transfer, uniformly dispersing the instantaneous impact stress over a broader area of the material to mitigate stress concentration. More critically, rigid microfibrils aligned in specific directions forced the main crack to undergo deflection (Crack deflection), requiring the crack to bypass the microfibrils along a tortuous, jagged path (Tortuous path) to continue propagating. This jagged path greatly increased the area of the newly generated fracture surface and was accompanied by the elastic deformation of the microfibrils and interfacial slip resistance. This efficiently dissipates external impact energy, endowing the material with excellent macroscopic fracture toughness.

### 3.4. Geometric Solidification Driven by Injection Thermal History

The preparation of in-situ microfibrillar composites relies on the precise matching of multi-stage processing temperatures and shear parameters. As detailed in Section 2.2, the first stage of melt blending was conducted at 275 °C with a screw speed of 150 rpm. These specific parameters are consistent with the general processing requirements for highly immiscible polymer blends. They ensured the complete melting of both components and provided an appropriate shear rate to achieve a moderate initial droplet size for the dispersed PA66 phase, establishing an ideal morphological foundation for the subsequent extensional drawing without causing excessive shear degradation.

In the second stage of extrusion drawing and fibrillation, the synergistic control of the barrel temperature and the secondary extrusion screw speed directly determined the quality and continuity of the microfibrils. Regarding temperature conditions (Table S4), if the barrel temperature was too low (210 °C), the surface quality of the die extrudate was poor, and the dispersed phase PA66 remained in a solid state, causing the melt to easily break during traction and drawing; if the temperature was too high (265 °C, above the melting point of PA66), although the dispersed phase could be elongated into fibrils, it was highly prone to thermodynamic retraction upon exiting the die, making it difficult to maintain a high aspect ratio. Therefore, 245 °C, a temperature between the melting points of the two phases, was selected as the secondary extrusion temperature. At this point, the extrudate had a smooth surface, and the dispersed phase was in an ideal semi-molten state, making it extremely easy to be stably drawn in the flow field. Regarding the screw speed (Table S5), when the speed was as low as 20 rpm, the die extrusion is too slow to effectively match the traction machine, and the strands were easily broken; when the speed was increased to 90 rpm, the material was extruded too quickly, leading to insufficient plasticization of the dispersed phase, resulting in rough extruded strands that were similarly prone to breakage during the drawing process. Therefore, the experiment finally established 35 rpm as the optimal screw speed. Under this condition, the extrusion was uniform and fully plasticized, and it could stably match the traction equipment to prepare microfibrillar strands with varying draw ratios. It is worth noting that in standard free-extrusion forming processes, significant extrudate swell (die swell) is inevitable due to the viscoelastic recovery of the polymer melt. However, our continuous drawing setup created a condition different from standard practice. While the tendency to swell was not entirely absent at the exact die exit, the immediate application of a strong uniaxial drawing force mechanically outpaced the macromolecular elastic transverse relaxation. Consequently, the macroscopic die swell was significantly mitigated rather than completely eliminated, which ensured stable processing and precise dimensional control of the microfibrils.

**Figure 11:**
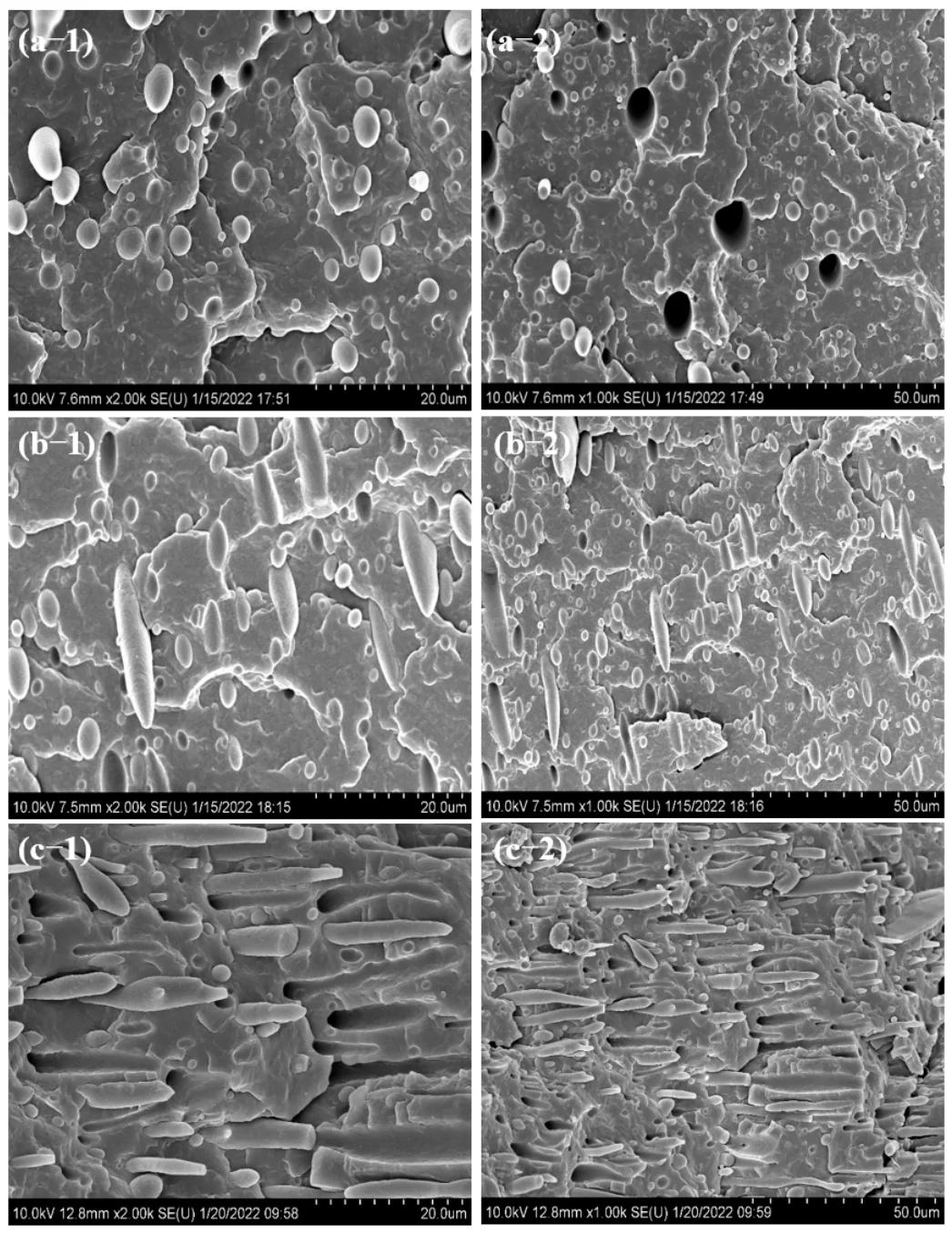
SEM images of PP/PA66 (85/15) at injection molding temperatures of (**a-1**,**a-2**) 265 °C, (**b-1**,**b-2**) 240 °C, and (**c-1**,**c-2**) 210 °C, respectively.

Although the aforementioned extrusion drawing process could impart a high-aspect-ratio morphology to the dispersed phase, this non-equilibrium microfibrillar system existed in a highly thermodynamic metastable state. The thermal processing history (injection molding temperature) of the final product played a decisive role in the retention of the microfibrillar morphology. By comparing the SEM morphologies of samples at different injection temperatures (high temperature 265 °C, medium temperature 240 °C, low temperature 210 °C) (Figure 11), significant structural evolution could be observed. When injected at 265 °C (higher than the PA66 melting point), the already elongated PA66 microfibrils underwent remelting; to minimize the interfacial free energy of the system, the molten fibers experienced Rayleigh instability breakage during the matrix relaxation process, rapidly shrinking back into spherical droplets. When injected at 240 °C, the microfibrils underwent partial thermodynamic shrinkage, gradually thickening and evolving into ellipsoidal shapes. However, when injected under 210 °C conditions (higher than the PP melting point but lower than the PA66 melting point), the morphology of the dispersed phase microfibrils was completely retained in the matrix, successfully achieving the “geometric freezing” of the microfibril network. This experimental result perfectly corroborates the theoretical mechanisms of blend instability previously reported by Jayanarayanan [19] and Kuzmanović [20], proving that bypassing the melting point of the dispersed phase is the key to preventing thermodynamic retraction during secondary processing.

**Figure 12:**
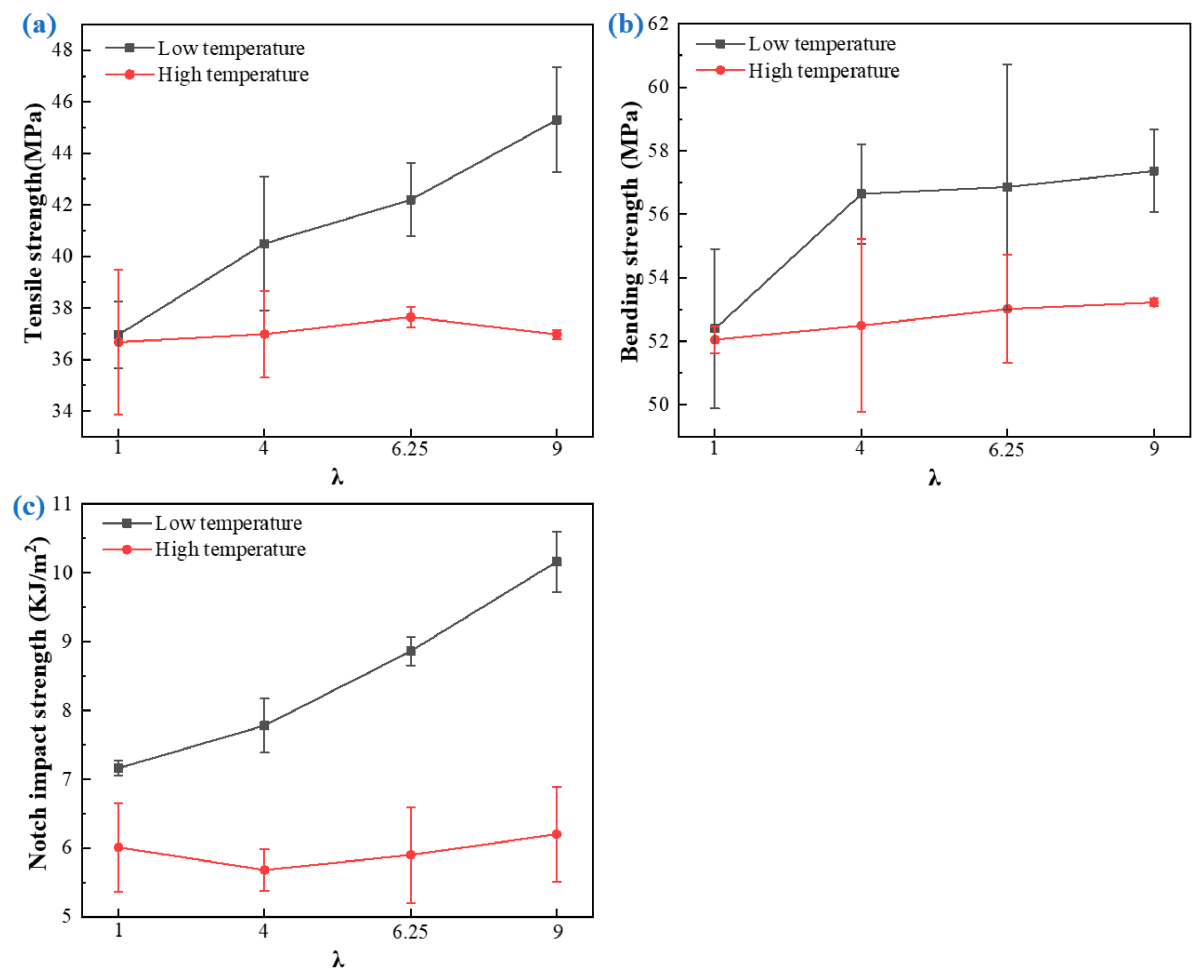
(**a**) Tensile strength, (**b**) Bending strength, (**c**) Notch impact strength of in-situ microfiber composite materials at different injection molding temperatures.

The evolution law of the microscopic morphology was directly confirmed by the macroscopic mechanical properties. Correlating the mechanical property test results (Figure 12) with the SEM microstructural observations (Figure 11) provided a clear understanding of the microfibers’ role during secondary processing. The data showed that all mechanical indicators of the high-temperature (265 °C) injection samples were significantly lower than those of the low-temperature (210 °C) injection samples, and essentially lost the reinforcement effect brought by the increase in the draw ratio (λ). Under high-temperature injection conditions, even after undergoing the maximum draw ratio (λ = 9) pulling beforehand, the tensile strength, flexural strength, and notched impact strength of its injection-molded parts were only 36.97 MPa, 53.23 MPa, and 6.20 kJ/m^2^, respectively, which were close to the performance of the undrawn (λ = 1) blends. This drastic mechanical degradation was in perfect agreement with the SEM morphological evidence in Figure 11(a-1,a-2). It confirmed that once the processing temperature exceeded the melting point of the dispersed phase, the high-aspect-ratio microfibers underwent Rayleigh instability and shrank back into spherical droplets, thereby completely losing their crucial roles in stress transfer and macroscopic tensile reinforcement.

Conversely, using the 210 °C low-temperature injection molding process, the microfibrillar morphology was solidified, and the mechanical properties of the composite material exhibited a significant positive response to the increase in the draw ratio. When λ = 9, the tensile strength of the 210 °C injected sample reached 45.3 MPa, the flexural strength reached 57.36 MPa, and the notched impact strength increased to 10.16 kJ/m^2^. This comparative result confirmed that strictly controlling the final injection processing temperature to be below the melting point of the dispersed phase is the critical boundary condition for ensuring the stable retention of the in-situ microfibrillar structure, effectively transferring the mechanical dividends imparted by the processing flow field to the final macroscopic components.

The fundamental scientific reason for this mechanical leap lay in the successful retention and uniform distribution of the microfibrillar network within the PP matrix. During low-temperature injection (210 °C), the PA66 fibrils remained in a solid, high-aspect-ratio state. This geometrically frozen network acted as a robust reinforcing skeleton. When tensile stress was applied, the load is efficiently transferred from the flexible PP matrix to the high-modulus PA66 microfibrils across the compatibilized interfaces. Furthermore, the uniformly distributed continuous fibrils restricted the mobility of the surrounding PP polymer chains and effectively dispersed local stress concentrations, preventing premature matrix yielding and significantly enhancing the macroscopic tensile strength of the composite.

### 3.5. Limitations and Future Research

Although this study systematically established and verified the complete cascade process window of “melt blending–hot drawing–low-temperature injection molding” for PP/PA66 in-situ microfibrillar composites, and thoroughly elucidated the mesoscopic mechanisms of morphological evolution and “geometric freezing”, deeper scientific questions still need to be addressed in the future due to space constraints and the current research focus. The core contribution of this paper lies in overcoming the morphological retention barrier of microfibrils during secondary molding from the perspectives of processing engineering and fracture mechanics, and successfully establishing a macroscopic structure–property relationship.

Therefore, it has not conducted an in-depth in-situ characterization of the molecular chain disentanglement and rearrangement dynamics at the two-phase interface under extreme extensional flow fields. Additionally, to provide a more intuitive visualization of the phase morphology and precise elemental distribution at the interfaces, advanced microscopic characterizations such as SEM-EDS, as well as in-depth FESEM fractography of the tensile fracture surfaces (e.g., to visualize fiber pull-out and interfacial debonding), will be systematically incorporated in our subsequent studies.

Future work will build upon the process boundaries established in this paper and utilize synchrotron radiation in-situ X-ray scattering (in-situ SAXS/WAXD) technology to real-time track the microscopic lamellar evolution and interfacial molecular dynamics of metastable microfibrils during their thermal shrinkage (Rayleigh instability) process. Simultaneously, a systematic quantitative measurement of the dispersed phase domain size and microfiber diameter is conducted to further elucidate the size dependent enhancement mechanism. Furthermore, considering the long-term service safety of high-performance engineering plastics in practical applications, the fatigue failure mechanisms and high-temperature creep resistance under complex alternating loads—which this paper has not touched upon—will also be focal points of upcoming research. These subsequent studies will rely on the dense microfibril network model successfully constructed in this paper, aiming to provide a more comprehensive cross-scale theoretical framework for the high-performance advancement of polyolefin composites.

## 4. Conclusions

In this study, PP/PA66 in-situ microfibrillar composites were successfully prepared through multi-stage thermal processing flow fields. The results show that there is a significant synergistic interactive effect between the extensional flow field and the interfacial compatibilizer during fibrillation: 3 wt% of PP-g-MAH provides the optimal balance between interfacial tension and viscous drag for fibrillation. Combined with a high draw ratio (λ = 9), it promotes the evolution of the PA66 dispersed phase from spherical shapes to highly oriented continuous microfibrils; whereas an excessive amount of compatibilizer (9 wt%) impedes deformation due to excessively strong interfacial adhesion, leading to hindered fibrillation. At the mesoscopic scale, the dense PA66 microfibril network profoundly alters the characteristics of the matrix. As a highly efficient heterogeneous nucleating agent, it significantly raises the crystallization temperature of pure PP (114.70 °C) to above 121 °C. However, it simultaneously acts as a physical barrier that restricts polymer chain folding, slightly lowering the overall crystallinity. Furthermore, the intertwined microfibrils construct a physical cross-linking network that exerts a strong confinement effect on the matrix chain segments. This causes a substantial increase in the low-frequency storage modulus and complex viscosity of the composite melt, exhibiting a pseudoplastic rheological characteristic that transitions from a liquid-like to a gel-like state.

Secondary processing thermal history has a decisive impact on the retention of the microfibrillar morphology. Conventional high-temperature injection molding (265 °C) triggers Rayleigh instability, causing the already elongated microfibrils to retract and break into spherical shapes, thereby losing the deformation dividends previously imparted by the flow field. In contrast, adopting a low-temperature injection molding strategy (210 °C), which lies between the melting points of the two phases, leverages the solid-state rigidity of the dispersed phase to successfully achieve the “geometric freezing” of the microfibrillar morphology, ensuring the efficient transfer of the mesoscopic structure to the macroscopic components.

Ultimately, the composite material achieved a leap in mechanical properties under the optimal processing window (15 wt% PA66, 3 wt% compatibilizer, λ = 9, 210 °C injection molding): the tensile strength, flexural strength, and notched impact strength reached 45.3 MPa, 57.36 MPa, and 10.16 kJ/m^2^, respectively. The highly oriented microfibrillar skeleton effectively overcomes the inherent defects of insufficient toughness and high notch sensitivity of the PP matrix by inducing main crack deflection and interfacial energy dissipation, while dynamically balancing the positive effects of fiber reinforcement against the increase in interfacial defects. This study provides quantitative experimental evidence for the structural regulation and engineering application of high-performance polyolefin microfibrillar composites.

## Data Availability

The original contributions presented in this study are included in the article/Supplementary Material. Further inquiries can be directed to the corresponding authors.

## Supplementary Materials

The following supporting information can be downloaded at: https://www.mdpi.com/article/10.3390/polym18172049/s1. Table S1: thermal properties and crystallinity of PP, PA66 and PP/PA66 Composites. Table S2: thermal properties of polymers in PP / PA66 in situ microfiber Composites. Table S3: Effect of Twin Screw Speed on Material Blending. Table S4: Effect of twin-screw processing temperature on PP/PA66 fiber stretching. Table S5: The Effect of Screw Speed on Extruded Stretch Wire. Table S6: Mechanical Properties of PP/PA66 (85/15) Microfiber Composite Materials under High and Low Temperature Injection Molding Conditions.

## References

[1] Obande W., Brádaigh C.M.Ó., Ray D. (2021). Continuous fibre-reinforced thermoplastic acrylic-matrix composites prepared by liquid resin infusion—A review. Compos. Part B Eng..

[2] Balogun O.A., Daramola O.O., Adediran A.A., Akinwande A.A., Bello O.S. (2023). Investigation of jute/tetracarpidium conophorum reinforced polypropylene composites for automobile application: Mechanical, wear and flow properties. Alex. Eng. J..

[3] Hossain M.T., Shahid M.A., Mahmud N., Habib A., Rana M.M., Khan S.A., Hossain M.D. (2024). Research and application of polypropylene: A review. Discov. Nano.

[4] Wang Y., Pan L., Li Z., Yue G., Man J. (2023). Study on static and dynamic mechanical properties of glass fiber reinforced polypropylene laminated self-reinforced polypropylene composite structure. Compos. Struct..

[5] Singh J., Kumar M., Kumar S., Mohapatra S.K. (2017). Properties of glass-fiber hybrid composites: A review. Polym.-Plast. Technol. Eng..

[6] Shubhra Q.T., Alam A., Quaiyyum M. (2013). Mechanical properties of polypropylene composites: A review. J. Thermoplast. Compos. Mater..

[7] Shen J., Wang M., Li J., Guo S. (2011). In situ fibrillation of polyamide 6 in isotactic polypropylene occurring in the laminating-multiplying die. Polym. Adv. Technol..

[8] Mishra R.K., Loganathan S., Thomas S. (2017). In-situ microfibrillar/nanofibrillar single polymer composites: Preparation, characterization, and application. Micro and Nano Fibrillar Composites (MFCs and NFCs) from Polymer Blends.

[9] Jiang J., Yang L., Jia C., Cao Y., Qiao Y., Hou J., Li Q., Liu X. (2023). Lightweight and high mechanical properties of in situ poly(ethylene terephthalate) reinforced polypropylene composite foams by chemical foam injection molding. Ind. Eng. Chem. Res..

[10] Chai J., Wang G., Wang S., Shao R., Zhao G. (2026). Overcoming strength-ductility trade-off in fully biodegradable polylactic acid/elastomer composites: Synergistic in situ topological fibrillation and supercritical CO_2_-induced nanocrystals. Macromolecules.

[11] Salehi A., Kheradmandkeysomi M., Rahmati R., Rahman S.S., Fashandi M., Park C.B. (2025). Tailoring interfacial, rheological, and energy absorption properties of in situ nanofibrillated ethylene propylene diene monomer rubber for enhanced toughness in polypropylene composites. ACS Appl. Polym. Mater..

[12] Li G., Fei Y., Kuang T., Liu T., Zhong M., Li Y., Jiang J., Turng L.-S., Chen F. (2022). The injected foaming study of polypropylene/multiwall carbon nanotube composite with in situ fibrillation reinforcement. Polymers.

[13] Wang Y., Li G., Wan Z., Zhu H., Ma Y., Xie L. (2024). Establishment of dynamic prediction model on morphology development of polypropylene/polyamide 6 blends under in-situ fibrillation flow. Polymer.

[14] Dadouche T., Yousfi M., Samuell C., Soulestin J., Lacrampe M.-F. The in-situ micro/nano-fibrillation concept as an efficient route for the enhancement of the processability and mechanical properties of bio-based polymeric blown films. Proceedings of the 35th International Conference of the Polymer Processing Society.

[15] Huang Y., He Y., Jiang L., Yang K., Xin C. (2016). Study on the processing of in situ fibrillation polypropylene_poly(hexamethylene adipamide) composites using a triple-screw. J. Beijing Univ. Chem. Technol..

[16] Huang Y., He Y., Ding W., Yang K., Yu D., Xin C. (2017). Improved viscoelastic, thermal, and mechanical properties of in situ microfibrillar polypropylene/polyamide 6,6 composites via direct extrusion using a triple-screw extruder. RSC Adv..

[17] Wang B., Sun C., Wang Y., Wang J., Yang Y., Cao Y., Song Y., Wang W. (2021). Improved mechanical properties of in situ microfibrillar polypropylene/polyamide6 composites through constructing strong interfacial adhesion. Polym. Adv. Technol..

[18] Cheng L., Wang J. (2018). Crystallization and morphological and crystal structures of PP in an in situ microfibrillar composite of modified PA66 with PP. Compos. Sci. Technol..

[19] Jayanarayanan K., Jose T., Thomas S., Joseph K. (2009). Effect of draw ratio on the microstructure, thermal, tensile and dynamic rheological properties of insitu microfibrillar composites. Eur. Polym. J..

[20] Kuzmanović M., Delva L., Cardon L., Ragaert K. (2016). The effect of injection molding temperature on the morphology and mechanical properties of PP/PET blends and microfibrillar composites. Polymers.

[21] Evstatiev M., Fakirov S. (1992). Microfibrillar reinforcement of polymer blends. Polymer.

[22] Evstatiev M., Schultz J.M., Petrovich S., Georgiev G., Fakirov S., Friedrich K. (1998). In situ polymer/polymer composites from poly(ethylene terephthalate), polyamide-6, and polyamide-66 blends. J. Appl. Polym. Sci..

[23] Liang D., Zhang Q., Du R., Fu Q. (2007). Morphology and mechanical properties of polypropylene with in situ formed polyamide-6 fibrils. Chin. J. Polym. Sci..

[24] (2006). Plastics-Determination of Tensile Properties-Part 2: Test Conditions for Moulding and Extrusion Plastics.

[25] (2008). Plastics-Determination of Flexural Properties.

[26] (2008). Plastics-Determination of Izod Impact Strength.

[27] Fu C., Niu K., Zhang R., Hua Z., Jiang L., Xue P. (2026). Material design and performance study of polyethylene vitrimer: Effects of crosslinking density and isomer structure. Adv. Eng. Mater..

[28] Tran N.H.A., Brünig H., Boldt R., Heinrich G. (2014). Morphology development from rod-like to nanofibrillar structures of dispersed poly (lactic acid) phase in a binary blend with poly (vinyl alcohol) matrix along the spinline. Polymer.

[29] Huang X., Li B., Shi B., Li L. (2008). Investigation on interfacial interaction of flame retarded and glass fiber reinforced PA66 composites by IGC/DSC/SEM. Polymer.

[30] (2000). Determination of the Melt Mass-Flow Rate (MFR) and the Melt Volume-Flow Rate (MVR) of Thermoplastics.

[31] Seo J., Kearney L.T., Datta S., Toomey M.D., Keum J.K., Naskar A.K. (2022). Tailoring compatibilization potential of maleic anhydride-grafted polypropylene by sequential rheochemical processing of polypropylene and polyamide 66 blends. Polym. Eng. Sci..

[32] Sacchi A., Di Landro L., Pegoraro M., Severini F. (2004). Morphology of isotactic polypropylene–polyamide 66 blends and their mechanical properties. Eur. Polym. J..

[33] Wailliez J., Regazzi P., Salonen A., Chen P.G., Jaeger M., Leonetti M., Rio E. (2024). Drop deformation in a planar elongational flow: Impact of surfactant dynamics. Soft Matter.

[34] Sundararaj U., Macosko C.W. (1995). Drop breakup and coalescence in polymer blends: The effects of concentration and compatibilization. Macromolecules.

