# Supplementary material for: Geometric Freezing of In-Situ Fibrillated PP/PA66 Composites via Low-Temperature Injection: Decoupling the Role of Draw Ratio and Compatibilization
Source: Polymers (Basel). 2026 Aug 24;18(17):2049. doi: 10.3390/polym18172049 (PMC13568407; doi:10.3390/polym18172049)
Supplement: Supplementary file 1 [file polymers-18-02049-s001.zip › polymers-4469357-supplementary.pdf]

Supporting Information

Geometric Freezing of In-Situ Fibrillated PP/PA66 Composites via Low-Temperature Injection: Decoupling the Role of Draw Ratio and Compatibilization

Run ZHANG[1,2], Chenchao Fu[2,], Guozheng Zhao[1], Weiheng Mo[1], Famin Zhao[1], Xiangrong Li[1], Qiuxu Chen[1,*], Lin Zhuo[1,*]*

1. Guangxi Zhuang Autonomous Region Construction Engineering Quality Testing Center Co., Ltd., Nanning 530005, China

[2] College of Mechanical and Electrical Engineering, Beijing University of Chemical Technology, Beijing, 100029, China

[*] E-mail: fuchenchao1017@163.com (C.F.); 18458082128@163.com (Q.C.); kfghzt@163.com (L.Z.)

Specific performance results of materials

Table S1 thermal properties and crystallinity of PP, PA66 and PP/PA66 Composites

Experimental sample	PP phase				PA66 phase			
	T_m	T_c	ΔH	X_c	T_m	T_c	ΔH	X_c
PP	162.40	114.70	116.50	55.69	—	—	—	—
PA66	—	—	—	—	260.60	226.70	91.60	47.05
PP/PA66(95/5)	162.31	121.21	87.81	44.18	259.30	234.10	4.52	46.44
PP/PA66(90/10)	162.70	122.53	76.82	40.82	258.00	234.40	8.94	45.89
PP/PA66(85/15)	162.82	122.01	85.41	48.03	258.30	234.10	12.31	42.15
PP/PA66(80/20)	164.14	121.51	89.24	53.32	259.40	233.60	16.44	42.22
PP/PA66(75/25)	163.61	121.44	83.42	53.17	258.50	232.60	19.55	40.17

Table S2 thermal properties of polymers in PP / PA66 in situ microfiber Composites

Experimental sample	PP phase						PA66 phase		
	T_m/℃	T_c/℃	△T_c	**ΔH**	T_g/℃	X_c	T_m/℃	T_c/℃	T_g/℃
85/15	162.82	121.51	41.30	85.41	-21.50	48.03	258.30	234.10	50.00
85/15/1	163.80	125.60	38.20	92.92	-21.80	52.78	258.30	234.50	50.50
85/15/3	163.70	125.90	37.80	87.94	-22.10	50.94	259.30	234.70	52.50

85/15/6	162.70	124.20	38.50	86.78	-23.60	51.73	258.00	233.40	58.30
85/15/9	163.00	125.00	38.00	95.94	-23.90	58.81	258.50	232.60	62.10

Table S3 Effect of Twin Screw Speed on Material Blending

Screw Speed (rpm)	Experimental Results
80	The discharge of the machine head is relatively slow, and the material can be completely melted. The two materials are mixed evenly, and the particle size of the dispersed phase is relatively large
150	The discharge from the machine head is uniform, and both materials are completely melted. The particle size of the dispersed phase is moderate and easy to form fibers
200	The discharge of the machine head is too fast and uneven, with some materials in a molten state and dispersed phase particles with too small size

Table S4 Effect of twin-screw processing temperature on PP/PA66 fiber stretching

Barrel Temperature (℃)	Experimental Results
210	The surface quality of the material extruded by the machine head is poor, the dispersed phase is in a solid state, and the melt is prone to breakage during the stretching process
245	The extruded material has a smooth surface and is dispersed in a semi molten state, making it easy to form fibers during the stretching process

265	The processing temperature is higher than the melting point temperature of the dispersed phase, and the dispersed phase is prone to shrinkage after forming fibers

Table S5 The Effect of Screw Speed on Extruded Stretch Wire

Screw Speed (rpm)	Experimental Results
20	The discharge speed of the machine head is too slow, which is not conducive to traction and stretching, and the extruded filament is prone to breakage
35	The material extruded by the machine head is relatively uniform, and the material is in a completely melted state. The traction speed can be adjusted to obtain splines with different stretching ratios
90	The extrusion of materials by the machine head is relatively fast, which is not conducive to the plasticization of dispersed phases. The extruded filaments are rough and prone to breakage during the stretching process

Table S6 Mechanical Properties of PP/PA66 (85/15) Microfiber Composite Materials under High and Low Temperature Injection Molding Conditions

	265℃			210℃		
Stretch Ratio	Tensile Strength /MPa	Bending Strength /MPa	Notch impact strength / kJ/m²	Tensile Strength /MPa	Bending Strength /MPa	Notch impact strength / kJ/m²
λ=1	36.67	52.05	6.01	36.96	52.4	7.16
λ=4	36.98	52.50	5.68	40.5	56.64	7.78
λ=6.25	37.65	53.02	5.90	42.2	56.86	8.86

λ=9	36.97	53.23	6.20	45.3	57.36	10.16
